# Submucous Resection of the Nasal Septum

**Published:** 1905-08

**Authors:** James J. Mooney

**Affiliations:** Buffalo, N. Y., Clinical Lecturer on Laryngology, Rhinology and Otology at the University of Buffalo; Laryngologist, Rhinologist and Otologist to the Buffalo Hospital of the Sisters of Charity, Emergency Hospital, Saint Mary’s Infant Asylum, etc.; 393 Seventh Street


### ORIGINAL COMMUNICATIONS.

### Submucous Resection of the Nasal Septum.

By JAMES J. MOONEY, M. D., Buffalo, N. Y.,

Clinical Lecturer on Laryngology, Rhinology and Otology at the University of Buffalo; Laryngologist, Rhinologist and Otologist to the Buffalo Hospital of the Sisters of Charity, Emergency Hospital, Saint Mary's Infant Asylum, etc.

OPERATIONS for the correction of septal deflections by the so-called straightening method are as many and varied as there are degrees of deviation, and this in itself is ample proof of the unsatisfactoriness of the procedures or their results.

To the patient suffering from the ordinary obstructive symptoms attendant upon a deflected septum, the prospect of having to wear a splint for from four to six weeks is by no means an alluring one, and as a consequence many go on suffering rather than undergo the discomforts of any of the straightening operations which, up to within a comparatively short time, were the only methods offering any prospect of relief.

While the operation is unsatisfactory to the patient primarily because of the splint, to the conscientious surgeon it is even more so, because of the uncertainty of its permanency as regards results; for, judging from a large personal experience, very many of these deflections recur to a greater or less degree after from one to two or three years, reproducing in proportion to the degree of recurrence the old obstructive symptoms, and bringing down upon the head of the unfortunate operator the censure of the patient.

Realising the uncertainty of permanent beneficial results, many men have, like myself, preferred to resort to every other expedient that gave promise of relief in even only partially restoring the patency of the part rather than to advise the major operation. In this direction all redundancies of the septum have been removed and portions of the turbinates ablated—which, all things being considered, was a justifiable sacrifice of important tissue.

To rhinologists who looked for results only and who were not responsible for any of the straightening operations with their multitudinous instruments and complex technic, the submucous resection of the offending septum, introduced by Killian, offered a means whereby practically all of the objections to the older methods were overcome. But even with all this there existed the tediousness and consequent nervous strain on both patient and operator due to the length of time required for the performance of the operation—being from half an hour to an hour. This was caused by the fact that after the initial incisions had been made, the mucous membrane elevated and the cartilage cut above and below with the knife devised by Killian, the removal had to be done piecemeal.

Killian's knife resembles a small tuning-fork in appearance, with a blade at its extremity extending across between the tines. The tines straddle the septum, and as the instrument is pressed backward the blade severs the cartilage.

At the recent meeting of the American Laryngological, Rhinological and Otological Society, held in Boston, Dr. M. L. Ballenger, of Chicago, presented an instrument which, though fashioned on exactly the same lines as Dr. Killian's is vastly superior to it in every way for, instead of having an immovable blade capable of cutting in but one direction, Dr. Ballenger conceived the idea of hanging it on a swivel so that no matter in what direction the instrument be pressed or swung it will cut in that direction. The advantages gained by this are very appreciable, for with one sweep the offending portion of the cartilaginous septum is severed on all sides and the entire mass removed in a very few seconds, thus considerably reducing the time required for the completion of the operation. Through this also the technic has been simplified to a great extent, as will be seen by the following description of the method of operating.

Both sides of the septum are first swabbed with a 20 per cent. solution of cocaine, after which adrenalin 1-1000 may be applied if desired. A semilunar incision is made through the mucous membrane and perichondrium—it matters not on which side, the operator choosing which ever is the more convenient; with a sharp elevator the membrane and perichondrium are lifted away from the cartilage for about an eighth or a quarter of an inch, great care being taken that the perichondrium is elevated or the membrane will be torn in the process of loosening. A dull elevator is then introduced and the membrane and perichondrium detached beyond the lines of the part to be removed.

The cartilage should then be cut through at the original semilunar incision, the finger being introduced into the opposite nostril, acting as a guide, in order that the membrane of that side be not cut through. The tip of the nose is then turned sharply aside and the cut end of the cartilage is brought into view. With the sharp elevator the loosening of the mucous membrane and perichondrium of the opposite side are started for a short distance, then the dull elevator is used to separate them well back, as was done on the other side. The Ballenger knife is then introduced and carried along the base of the cartilage to its posterior end, then swung upward, forward and finally downward to the starting point, when the entire mass can be taken with forceps through the original opening in the mucous membrane—which, by the way, need not be over half an inch in length. Should there be any spurs or ridges they may now be removed, submucously, through this opening, with the chisel or gouge. The cut edges of membrane fall together, a light gauze packing is introduced, and the operation is complete. Ordinarily, in from twenty-four to forty-eight hours healing will take place by first intention. The procedure from the initial incision to the packing with gauze should not ordinarily consume more than from four to six minutes. There is no pain and no hemorrhage.

To those who have been doing any of the straightening operations, or who later took up the Killian method of resection, the advantages of this operation will appeal strongly, for it approaches very nearly the ideal both from the patient's and surgeon's standpoint.

Over the Killian method it has the advantages of simplicity of technic, shortness of time required and the fact that the operation may be done on the left side always, or the right, regardless of the direction of the deflection, thereby enabling the operator to choose that side which is handiest for him upon which to work.

It is hardly necessary to enter into detail as to the advantages of submucous resection over the straightening operations further than to state that with this procedure there can be no recurrence and that is, after all, the main consideration; while the doing away with the splint and the shortening of the healing process to a period of from twenty-four to forty-eight hours are advantages which cannot be overestimated.

That this method is applicable to all varieties of septal deflections is not claimed, but that it will supplant the older methods in the vast majority of cases is a certainty—and rhinologists will feel that they can offer to patients suffering from this trouble assurances of lasting beneficial results, which they could not conscientiously do previous to the introduction of operations for the submucous resection of the nasal septum.

393 Seventh Street.

---

The Making of an M. D.—Bacon—Men don't become doctors all at once, do they?

Egbert—No, by degrees.—*Yonkers Statesman.*

